# Bis{μ_2_-2-[(2-hy­droxy­eth­yl)(meth­yl)amino]­ethano­lato}bis­(μ_3_-*N*-methyl-2,2′-aza­nediyldiethano­lato)tetra­kis­(thio­cyan­atato-κ*N*)dichromium(III)dimanganese(II) dimethyl­formamide tetra­solvate

**DOI:** 10.1107/S1600536811049336

**Published:** 2011-11-30

**Authors:** Valentyna V. Semenaka, Oksana V. Nesterova, Volodymyr N. Kokozay, Roman I. Zubatyuk, Oleg V. Shishkin

**Affiliations:** a Department of Inorganic Chemistry,Taras Shevchenko National University of Kyiv, Volodymyrs’ka St. 64, Kyiv 01601, Ukraine; b STC "Institute for Single Crystals" National Academy of Sciences of Ukraine, 60, Lenina Avenue, Kharkiv 61001, Ukraine

## Abstract

The heterometallic title complex, [Cr_2_Mn_2_(C_5_H_11_NO_2_)_2_(C_5_H_12_NO_2_)_2_(NCS)_4_]·4C_3_H_7_NO, was prepared using manganese powder, Reineckes salt, ammonium thio­cyanate and a non-aqueous solution of *N*-methyl­diethano­lamine in air. The centrosymmetric mol­ecular structure of the complex is based on a tetra­nuclear {Mn_2_Cr_2_(μ-O)_6_} core. The tetra­nuclear complex mol­ecule and the two uncoordinated dimethyl­formamide mol­ecules are linked by O—H⋯O hydrogen bonds, while the two other mol­ecules of dimethyl­formamide do not participate in hydrogen bonding.

## Related literature

For background to polynuclear chromium-containing complexes, see: McInnes *et al.* (2005 ▶); Affronte *et al.* (2005 ▶). For the use of amino ­alcohols with versatile bridging modes in generating such metal clusters, see: Langley *et al.* (2009 ▶); Ferguson *et al.* (2008 ▶); Saalfrank *et al.* (2001 ▶). For background to direct synthesis, see: Kokozay & Shevchenko (2005 ▶). 
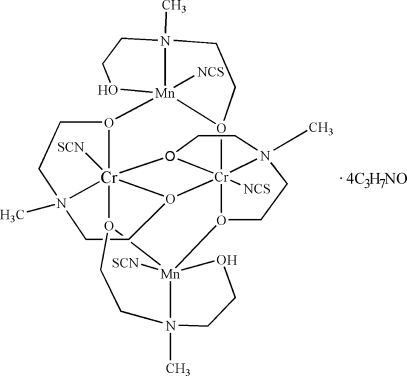


## Experimental

### 

#### Crystal data

[Cr_2_Mn_2_(C_5_H_11_NO_2_)_2_(C_5_H_12_NO_2_)_2_(NCS)_4_]·4C_3_H_7_NO
*M* *_r_* = 1208.64
Monoclinic, 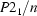

*a* = 11.5207 (2) Å
*b* = 13.5261 (2) Å
*c* = 18.5825 (4) Å
β = 106.123 (2)°
*V* = 2781.81 (9) Å^3^
*Z* = 2
Mo *K*α radiation
μ = 1.04 mm^−1^
*T* = 100 K
0.3 × 0.2 × 0.1 mm

#### Data collection

Oxford Diffraction Xcalibur Sapphire3 diffractometer
Absorption correction: multi-scan (*CrysAlis RED*; Oxford Diffraction, 2008 ▶) *T*_min_ = 0.6, *T*_max_ = 0.8
14864 measured reflections
8065 independent reflections
5070 reflections with *I* > 2σ(*I*)
*R*_int_ = 0.025

#### Refinement

*R*[*F* ^2^ > 2σ(*F* ^2^)] = 0.032
*wR*(*F* ^2^) = 0.048
*S* = 0.98
8065 reflections
313 parameters
H-atom parameters constrained
Δρ_max_ = 0.43 e Å^−3^
Δρ_min_ = −0.38 e Å^−3^

### 

Data collection: *CrysAlis PRO* (Oxford Diffraction, 2010 ▶); cell refinement: *CrysAlis PRO*; data reduction: *CrysAlis PRO*; program(s) used to solve structure: *SHELXS97* (Sheldrick, 2008 ▶); program(s) used to refine structure: *SHELXL97* (Sheldrick, 2008 ▶); molecular graphics: *PLATON* (Spek, 2009 ▶); software used to prepare material for publication: *publCIF* (Westrip, 2010 ▶).

## Supplementary Material

Crystal structure: contains datablock(s) I, global. DOI: 10.1107/S1600536811049336/zk2033sup1.cif

Structure factors: contains datablock(s) I. DOI: 10.1107/S1600536811049336/zk2033Isup2.hkl

Supplementary material file. DOI: 10.1107/S1600536811049336/zk2033Isup3.cdx

Additional supplementary materials:  crystallographic information; 3D view; checkCIF report

## Figures and Tables

**Table 1:** Hydrogen-bond geometry (Å, °)

*D*—H⋯*A*	*D*—H	H⋯*A*	*D*⋯*A*	*D*—H⋯*A*
O3—H3⋯O5	0.82	1.78	2.5985 (18)	176

**Fig. 1.**
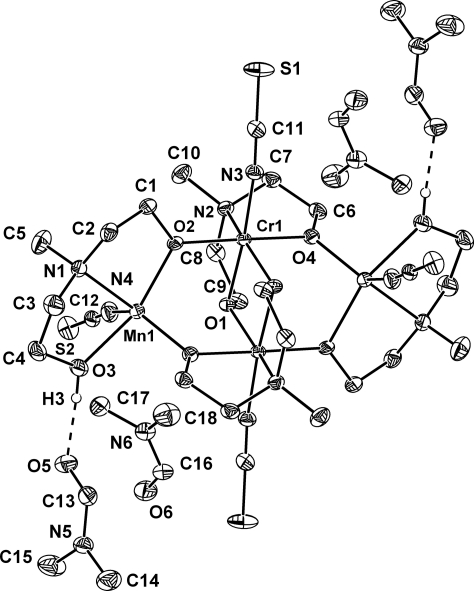
Molecular structure of the complex, showing the atom numbering, with 50% probability displacement ellipsoids

## supplementary crystallographic information

### Comment

Great interest in the synthesis and investigation of polynuclear chromium- and manganese-containing compounds dates from the late 90 s mostly due to the works R.E.P. Winpenny and coworkers devoted to magnetic studies of high-nuclear cages and wheels (McInnes *et al.*, 2005; Affronte *et al.*, 2005). At the same time, the potential of alcohols and amino alcohols in generating such metal clusters was widely explored (Saalfrank *et al.*, 2001; Langley *et al.*, 2009; Ferguson *et al.*, 2008).The polydentate alkoxo ligands possessing versatile bridging modes were recognized as promising reagents for synthesis of new heterometallic complexes. Previously we have demonstrated that amino alcohols represent a powerful tool for assembling polynuclear metal complexes in conditions of the synthetic approach named "direct synthesis of coordination compounds". This strategy employs metal powders or metal oxides as starting materials and eliminates the separate step of building block construction, proving to be an efficient route to obtain new heterometallic complexes (Kokozay & Shevchenko, 2005). Novel heterometallic compound [Mn_2_Cr_2_(NCS)_4_(HMeDea)_2_(MeDea)_2_]^.^4dmf have been prepared in one-step self-assembly reaction of zerovalent manganese, Reineckes salt, ammonium thiocyanate and dymethylformamide (dmf) solution of *N*-methyldiethanolamine (H_2_MeDea) in air using molar ratio Mn^0^:NH_4_[Cr(NCS)_4_(NH_3_)_2_]^.^H_2_O = 4: 1. X-ray diffraction studies reveal that the centrosymmetric molecular structure of the complex is based on a tetranuclear {Mn_2_Cr_2_(µ-O)_6_} core with the metal atoms arranged in a planar rhombic array. In the present complex MeDea and HMeDea ligands adopt a chelating-bridging mode forming five-membered rings. Both manganese(II) ions are five coordinated and have N_2_O_3_ donor sets (Fig. 1) formed by three oxygen and one nitrogen atom of *N*-methyldiethanolamine ligands and one nitrogen atom of terminal thiocyanate group. The Mn–O(N) bond lengths vary in the range 2.0651 (10)–2.3004 (13) Å, while *cis* and *trans* O(N)–Mn–O(N) bond angles range from 65.28 (6)° to 124.35 (14)° and from 140.06 (9)° to 173.0 (3)°, respectively. Each chromium(III) atom has distorted octahedral environment comprised by four oxygen and one nitrogen atoms from *N*-methyldiethanolamine ligands and one nitrogen atom from terminal thiocyanate group. The Cr–O(N) distances are in the range of 1.9391 (10)–2.0974 (14) Å. The *cis* and *trans* O(N)–Cr–O(N) bond angles vary from 80.70 (5)° to 101.65 (5)° and from 158.59 (5)° to 177.32 (5)°, respectively. Tetranuclear molecule of the complex and two dmf molecules are linked together by O–H···O hydrogen bonds [O(3)–H(3)···O(5): D–A = 2.598 Å, D–H···A = 175.99°], two other uncoordinated molecules of dmf are not involved in hydrogen bonding.

### Experimental

Manganese powder (0.137 g, 2.5 mmol), NH_4_[Cr(NCS)_4_(NH_3_)_2_]^.^H_2_O (0.221 g, 0.625 mmol), NH_4_NCS (0.333 g, 4.375 mmol), dmf (20 mL) and *N*-methyldiethanolamine (0.80 cm^3^) were heated to 50–60° and stirred magnetically during 2 h. Dark blue crystals suitable for the X-ray crystallographic study were deposited after several months after addition of diethyl ether and Pr^i^OH into the resulting blue solution. The crystals were filtered off, washed with dry Pr^i^OH, and finally dried at room temperature. Yield: 0.09 g, 24% (per chromium). Anal. Calc. for C_36_H_74_Mn_2_Cr_2_N_12_O_12_S_4_ (*M* = 1208.64): Mn, 9.09; Cr, 8.60; C, 35.78; H, 6.12; N, 13.91; S, 10.61. Found: Mn, 9.1; Cr, 8.8; C, 35.8; H, 6.2; N, 13.8; S, 10.7. IR: 2889(*m*), 2867(sh), 2818(sh), 2080(*vs*), 1660(*s*), 1458(w), 1449(sh), 1410(sh), 1383(*m*), 1355(w), 1308(w), 1260(sh), 1253(w), 1207(sh), 1171(sh), 1143(sh), 1075(*s*), 1032(sh), 1002(sh), 980(sh), 913(*m*), 764(sh), 744(*m*), 676(*m*), 643(sh), 545(*m*), 517(*m*), 474(w), 419(sh), 412(w). The compound is sparingly soluble in dmso and dmf, insoluble in water and it is indefinitely stable in air.

### Refinement

All non-hydrogen atoms were located from the initial solution and refined with anisotropic thermal parameters. The hydrogen atoms were positioned geometrically and included into refinement using riding model approximation with *U*_iso_=nUeq of non-hydrogen carrier atom (n = 1.5 for CH_3_ and OH groups and n = 1.2 for remaining H-atoms)

### Crystal data

**Table:**

[Cr_2_Mn_2_(C_5_H_11_NO_2_)_2_(C_5_H_12_NO_2_)_2_(NCS)_4_]·4C_3_H_7_NO	*Z* = 2
*M**_r_* = 1208.64	*F*(000) = 1264
Monoclinic, *P*2_1_/*n*	*D*_x_ = 1.443 Mg m^−^^3^
*a* = 11.5207 (2) Å	Mo *K*α radiation, λ = 0.71073 Å
*b* = 13.5261 (2) Å	µ = 1.04 mm^−^^1^
*c* = 18.5825 (4) Å	*T* = 100 K
β = 106.123 (2)°	Block, dark blue
*V* = 2781.81 (9) Å^3^	0.3 × 0.2 × 0.1 mm

### Data collection

**Table:**

Oxford Diffraction Xcalibur Sapphire3 diffractometer	5070 reflections with *I* > 2σ(*I*)
graphite	*R*_int_ = 0.025
ω scans	θ_max_ = 30°, θ_min_ = 2.9°
Absorption correction: multi-scan (*CrysAlis RED*; Oxford Diffraction, 2008)	*h* = −12→16
*T*_min_ = 0.6, *T*_max_ = 0.8	*k* = −16→19
14864 measured reflections	*l* = −26→12
8065 independent reflections	

### Refinement

**Table:**

Refinement on *F*^2^	Primary atom site location: structure-invariant direct methods
Least-squares matrix: full	Secondary atom site location: difference Fourier map
*R*[*F*^2^ > 2σ(*F*^2^)] = 0.032	Hydrogen site location: inferred from neighbouring sites
*wR*(*F*^2^) = 0.048	H-atom parameters constrained
*S* = 0.98	*w* = 1/[σ^2^(*F*_o_^2^) + (0.010*P*)^2^] where *P* = (*F*_o_^2^ + 2*F*_c_^2^)/3
8065 reflections	(Δ/σ)_max_ = 0.002
313 parameters	Δρ_max_ = 0.43 e Å^−^^3^
0 restraints	Δρ_min_ = −0.38 e Å^−^^3^

### Special details

**Table:**

Experimental. CrysAlis RED, Oxford Diffraction Ltd., Version 1.171.32.24 (release 21-04-2008 CrysAlis171 .NET) (compiled Apr 21 2008,18:23:10) Empirical absorption correction using spherical harmonics, implemented in SCALE3 ABSPACK scaling algorithm.
Geometry. All e.s.d.'s (except the e.s.d. in the dihedral angle between two l.s. planes) are estimated using the full covariance matrix. The cell e.s.d.'s are taken into account individually in the estimation of e.s.d.'s in distances, angles and torsion angles; correlations between e.s.d.'s in cell parameters are only used when they are defined by crystal symmetry. An approximate (isotropic) treatment of cell e.s.d.'s is used for estimating e.s.d.'s involving l.s. planes.
Refinement. Refinement of *F*^2^ against ALL reflections. The weighted *R*-factor *wR* and goodness of fit *S* are based on *F*^2^, conventional *R*-factors *R* are based on *F*, with *F* set to zero for negative *F*^2^. The threshold expression of *F*^2^ > σ(*F*^2^) is used only for calculating *R*-factors(gt) *etc*. and is not relevant to the choice of reflections for refinement. *R*-factors based on *F*^2^ are statistically about twice as large as those based on *F*, and *R*- factors based on ALL data will be even larger.

### Fractional atomic coordinates and isotropic or equivalent isotropic displacement parameters (Å^2^)

**Table:**

	*x*	*y*	*z*	*U*_iso_*/*U*_eq_	
Mn1	0.50236 (2)	0.224755 (17)	0.513287 (14)	0.01778 (6)	
Cr1	0.38351 (2)	−0.005009 (19)	0.523254 (14)	0.01505 (6)	
S1	0.16877 (4)	−0.10240 (4)	0.69652 (3)	0.04214 (14)	
S2	0.25156 (4)	0.38492 (3)	0.28877 (2)	0.02974 (11)	
O1	0.44591 (9)	0.05372 (7)	0.44303 (6)	0.0156 (2)	
O2	0.41428 (9)	0.12490 (7)	0.56870 (6)	0.0181 (2)	
O3	0.62650 (11)	0.35388 (8)	0.52249 (7)	0.0325 (3)	
H3	0.6726	0.3704	0.4981	0.049*	
O4	0.34983 (9)	−0.13296 (7)	0.47288 (6)	0.0181 (2)	
O5	0.77327 (11)	0.41465 (9)	0.44798 (7)	0.0340 (3)	
O6	0.56001 (11)	0.24649 (9)	0.10559 (7)	0.0342 (3)	
N1	0.48650 (11)	0.31521 (9)	0.61496 (7)	0.0197 (3)	
N2	0.21359 (11)	0.02768 (9)	0.45005 (7)	0.0189 (3)	
N3	0.31690 (12)	−0.05439 (10)	0.60560 (8)	0.0218 (3)	
N4	0.38177 (13)	0.28738 (10)	0.41875 (8)	0.0270 (3)	
N5	0.95472 (13)	0.40818 (10)	0.42324 (8)	0.0271 (4)	
N6	0.55036 (12)	0.27485 (11)	0.22479 (8)	0.0262 (3)	
C1	0.38489 (15)	0.16079 (12)	0.63305 (9)	0.0242 (4)	
H1A	0.3877	0.1059	0.6688	0.029*	
H1B	0.302	0.1882	0.6186	0.029*	
C2	0.47414 (15)	0.24061 (12)	0.66996 (9)	0.0234 (4)	
H2A	0.4462	0.2729	0.7099	0.028*	
H2B	0.5539	0.2102	0.6933	0.028*	
C3	0.59968 (16)	0.37190 (14)	0.64366 (10)	0.0316 (4)	
H3A	0.6658	0.3265	0.6689	0.038*	
H3B	0.5894	0.421	0.6809	0.038*	
C4	0.63246 (17)	0.42432 (13)	0.58016 (10)	0.0333 (5)	
H4A	0.5753	0.4792	0.5611	0.04*	
H4B	0.715	0.4521	0.5978	0.04*	
C5	0.38177 (16)	0.38211 (13)	0.59616 (10)	0.0347 (5)	
H5A	0.38	0.4204	0.6406	0.052*	
H5B	0.3074	0.3433	0.5792	0.052*	
H5C	0.3882	0.4272	0.5562	0.052*	
C6	0.23263 (14)	−0.14850 (12)	0.42388 (9)	0.0212 (4)	
H6A	0.2025	−0.2145	0.4331	0.025*	
H6B	0.2358	−0.146	0.3712	0.025*	
C7	0.14866 (14)	−0.06917 (11)	0.43730 (10)	0.0226 (4)	
H7A	0.0769	−0.0642	0.3934	0.027*	
H7B	0.1209	−0.0865	0.4816	0.027*	
C8	0.23655 (14)	0.06676 (12)	0.37959 (9)	0.0227 (4)	
H8A	0.2299	0.1397	0.3791	0.027*	
H8B	0.1737	0.041	0.3358	0.027*	
C9	0.35990 (13)	0.03810 (12)	0.37239 (9)	0.0200 (4)	
H9A	0.36	−0.0322	0.3577	0.024*	
H9B	0.3806	0.079	0.3335	0.024*	
C10	0.14028 (14)	0.09998 (13)	0.47868 (10)	0.0282 (4)	
H10A	0.0638	0.1119	0.4403	0.042*	
H10B	0.1849	0.1623	0.4907	0.042*	
H10C	0.1237	0.0736	0.5239	0.042*	
C11	0.25484 (15)	−0.07453 (12)	0.64353 (9)	0.0217 (4)	
C12	0.32760 (15)	0.32850 (12)	0.36446 (9)	0.0208 (4)	
C13	0.87761 (16)	0.38143 (13)	0.46022 (10)	0.0280 (4)	
H13A	0.9033	0.3336	0.4988	0.034*	
C14	1.07644 (16)	0.36743 (15)	0.44205 (12)	0.0428 (5)	
H14A	1.0877	0.3222	0.4846	0.064*	
H14B	1.1354	0.4213	0.4554	0.064*	
H14C	1.0881	0.3314	0.3988	0.064*	
C15	0.92280 (18)	0.48139 (15)	0.36406 (12)	0.0452 (6)	
H15A	0.8354	0.4931	0.3504	0.068*	
H15B	0.9458	0.4573	0.3202	0.068*	
H15C	0.9656	0.5433	0.3816	0.068*	
C16	0.59011 (15)	0.29153 (13)	0.16508 (10)	0.0283 (4)	
H16A	0.6473	0.3433	0.169	0.034*	
C17	0.46767 (16)	0.19439 (13)	0.22546 (10)	0.0310 (4)	
H17A	0.4335	0.1699	0.1743	0.047*	
H17B	0.4024	0.2181	0.2454	0.047*	
H17C	0.5112	0.1408	0.2572	0.047*	
C18	0.59935 (17)	0.32815 (15)	0.29436 (11)	0.0434 (5)	
H18A	0.6562	0.3785	0.2872	0.065*	
H18B	0.6415	0.2818	0.3335	0.065*	
H18C	0.5334	0.3601	0.3094	0.065*	

### Atomic displacement parameters (Å^2^)

**Table:**

	*U*^11^	*U*^22^	*U*^33^	*U*^12^	*U*^13^	*U*^23^
Mn1	0.02102 (13)	0.01665 (12)	0.01768 (13)	−0.00053 (12)	0.00874 (10)	−0.00076 (11)
Cr1	0.01441 (12)	0.01699 (13)	0.01527 (13)	−0.00159 (12)	0.00662 (10)	−0.00100 (11)
S1	0.0342 (3)	0.0666 (4)	0.0332 (3)	−0.0060 (3)	0.0221 (2)	0.0104 (3)
S2	0.0363 (3)	0.0302 (3)	0.0181 (2)	−0.0017 (2)	−0.00015 (19)	0.0021 (2)
O1	0.0148 (6)	0.0195 (6)	0.0124 (6)	−0.0011 (5)	0.0040 (4)	0.0002 (5)
O2	0.0213 (6)	0.0202 (6)	0.0160 (6)	−0.0023 (5)	0.0106 (5)	−0.0040 (5)
O3	0.0441 (8)	0.0250 (7)	0.0377 (8)	−0.0126 (6)	0.0269 (7)	−0.0112 (6)
O4	0.0160 (6)	0.0192 (6)	0.0191 (6)	−0.0028 (5)	0.0049 (5)	−0.0033 (5)
O5	0.0265 (7)	0.0378 (8)	0.0414 (8)	0.0010 (6)	0.0158 (6)	0.0019 (7)
O6	0.0396 (8)	0.0382 (8)	0.0284 (7)	0.0000 (6)	0.0153 (6)	−0.0026 (6)
N1	0.0210 (8)	0.0180 (7)	0.0205 (7)	0.0010 (6)	0.0065 (6)	−0.0007 (6)
N2	0.0151 (7)	0.0205 (7)	0.0218 (8)	−0.0003 (6)	0.0066 (6)	−0.0022 (6)
N3	0.0243 (8)	0.0235 (8)	0.0202 (8)	−0.0041 (7)	0.0108 (6)	−0.0015 (6)
N4	0.0353 (9)	0.0211 (8)	0.0254 (8)	0.0013 (7)	0.0098 (7)	0.0021 (7)
N5	0.0265 (8)	0.0276 (8)	0.0304 (9)	0.0025 (7)	0.0131 (7)	0.0070 (7)
N6	0.0246 (8)	0.0296 (8)	0.0253 (8)	0.0001 (7)	0.0086 (6)	−0.0018 (7)
C1	0.0336 (10)	0.0234 (9)	0.0217 (9)	−0.0031 (8)	0.0176 (8)	−0.0055 (8)
C2	0.0302 (10)	0.0244 (9)	0.0167 (9)	0.0027 (8)	0.0086 (7)	−0.0029 (7)
C3	0.0352 (11)	0.0326 (10)	0.0270 (10)	−0.0097 (9)	0.0087 (8)	−0.0085 (9)
C4	0.0452 (12)	0.0240 (10)	0.0362 (12)	−0.0136 (9)	0.0204 (10)	−0.0102 (9)
C5	0.0431 (12)	0.0319 (11)	0.0299 (11)	0.0153 (10)	0.0117 (9)	0.0025 (9)
C6	0.0199 (9)	0.0221 (9)	0.0213 (9)	−0.0064 (8)	0.0050 (7)	−0.0028 (8)
C7	0.0160 (9)	0.0265 (9)	0.0254 (10)	−0.0052 (8)	0.0059 (7)	−0.0030 (8)
C8	0.0206 (9)	0.0239 (9)	0.0206 (9)	−0.0017 (8)	0.0006 (7)	0.0026 (8)
C9	0.0202 (9)	0.0249 (9)	0.0139 (8)	−0.0039 (7)	0.0031 (7)	0.0016 (7)
C10	0.0190 (9)	0.0316 (10)	0.0338 (11)	0.0051 (8)	0.0066 (8)	−0.0047 (9)
C11	0.0222 (9)	0.0235 (9)	0.0182 (9)	−0.0029 (8)	0.0038 (7)	0.0016 (7)
C12	0.0257 (10)	0.0178 (9)	0.0213 (9)	−0.0037 (8)	0.0105 (8)	−0.0031 (8)
C13	0.0320 (11)	0.0252 (10)	0.0284 (10)	−0.0016 (9)	0.0108 (8)	−0.0002 (8)
C14	0.0319 (12)	0.0445 (13)	0.0566 (15)	0.0105 (10)	0.0198 (10)	0.0128 (11)
C15	0.0407 (12)	0.0525 (14)	0.0455 (14)	0.0039 (11)	0.0173 (10)	0.0217 (11)
C16	0.0232 (10)	0.0303 (11)	0.0354 (11)	0.0013 (8)	0.0151 (8)	0.0000 (9)
C17	0.0341 (11)	0.0305 (10)	0.0316 (11)	0.0012 (9)	0.0143 (9)	0.0048 (9)
C18	0.0436 (13)	0.0519 (14)	0.0351 (12)	−0.0069 (11)	0.0119 (10)	−0.0138 (11)

### Geometric parameters (Å, °)

**Table:**

Mn1—O4^i^	2.0651 (10)	C1—H1B	0.99
Mn1—N4	2.0923 (15)	C2—H2A	0.99
Mn1—O2	2.1199 (10)	C2—H2B	0.99
Mn1—O3	2.2337 (11)	C3—C4	1.512 (2)
Mn1—N1	2.3004 (13)	C3—H3A	0.99
Cr1—O2	1.9391 (10)	C3—H3B	0.99
Cr1—O4	1.9546 (10)	C4—H4A	0.99
Cr1—O1	1.9914 (10)	C4—H4B	0.99
Cr1—O1^i^	2.0013 (10)	C5—H5A	0.98
Cr1—N3	2.0071 (13)	C5—H5B	0.98
Cr1—N2	2.0974 (14)	C5—H5C	0.98
Cr1—Cr1^i^	3.0428 (4)	C6—C7	1.511 (2)
S1—C11	1.6240 (16)	C6—H6A	0.99
S2—C12	1.6260 (18)	C6—H6B	0.99
O1—C9	1.4238 (18)	C7—H7A	0.99
O1—Cr1^i^	2.0013 (10)	C7—H7B	0.99
O2—C1	1.4161 (16)	C8—C9	1.514 (2)
O3—C4	1.4212 (19)	C8—H8A	0.99
O3—H3	0.8197	C8—H8B	0.99
O4—C6	1.4189 (18)	C9—H9A	0.99
O4—Mn1^i^	2.0651 (10)	C9—H9B	0.99
O5—C13	1.2437 (18)	C10—H10A	0.98
O6—C16	1.225 (2)	C10—H10B	0.98
N1—C5	1.470 (2)	C10—H10C	0.98
N1—C2	1.4710 (19)	C13—H13A	0.95
N1—C3	1.479 (2)	C14—H14A	0.98
N2—C10	1.4841 (18)	C14—H14B	0.98
N2—C7	1.4944 (19)	C14—H14C	0.98
N2—C8	1.5021 (19)	C15—H15A	0.98
N3—C11	1.1671 (17)	C15—H15B	0.98
N4—C12	1.169 (2)	C15—H15C	0.98
N5—C13	1.3159 (19)	C16—H16A	0.95
N5—C15	1.449 (2)	C17—H17A	0.98
N5—C14	1.456 (2)	C17—H17B	0.98
N6—C16	1.332 (2)	C17—H17C	0.98
N6—C17	1.449 (2)	C18—H18A	0.98
N6—C18	1.451 (2)	C18—H18B	0.98
C1—C2	1.517 (2)	C18—H18C	0.98
C1—H1A	0.99		
			
O4^i^—Mn1—N4	132.86 (5)	N1—C3—H3B	109.6
O4^i^—Mn1—O2	92.63 (4)	C4—C3—H3B	109.6
N4—Mn1—O2	111.71 (5)	H3A—C3—H3B	108.1
O4^i^—Mn1—O3	88.41 (4)	O3—C4—C3	107.69 (13)
N4—Mn1—O3	90.54 (5)	O3—C4—H4A	110.2
O2—Mn1—O3	147.52 (4)	C3—C4—H4A	110.2
O4^i^—Mn1—N1	117.93 (5)	O3—C4—H4B	110.2
N4—Mn1—N1	106.77 (5)	C3—C4—H4B	110.2
O2—Mn1—N1	77.41 (4)	H4A—C4—H4B	108.5
O3—Mn1—N1	73.53 (4)	N1—C5—H5A	109.5
O2—Cr1—O4	177.32 (5)	N1—C5—H5B	109.5
O2—Cr1—O1	84.50 (4)	H5A—C5—H5B	109.5
O4—Cr1—O1	93.42 (4)	N1—C5—H5C	109.5
O2—Cr1—O1^i^	96.72 (4)	H5A—C5—H5C	109.5
O4—Cr1—O1^i^	84.58 (4)	H5B—C5—H5C	109.5
O1—Cr1—O1^i^	80.70 (5)	O4—C6—C7	109.13 (13)
O2—Cr1—N3	91.83 (5)	O4—C6—H6A	109.9
O4—Cr1—N3	90.19 (5)	C7—C6—H6A	109.9
O1—Cr1—N3	175.88 (5)	O4—C6—H6B	109.9
O1^i^—Cr1—N3	101.65 (5)	C7—C6—H6B	109.9
O2—Cr1—N2	96.68 (5)	H6A—C6—H6B	108.3
O4—Cr1—N2	81.41 (5)	N2—C7—C6	109.47 (12)
O1—Cr1—N2	84.05 (4)	N2—C7—H7A	109.8
O1^i^—Cr1—N2	158.59 (5)	C6—C7—H7A	109.8
N3—Cr1—N2	94.53 (5)	N2—C7—H7B	109.8
O2—Cr1—Cr1^i^	90.82 (3)	C6—C7—H7B	109.8
O4—Cr1—Cr1^i^	88.67 (3)	H7A—C7—H7B	108.2
O1—Cr1—Cr1^i^	40.47 (3)	N2—C8—C9	112.56 (13)
O1^i^—Cr1—Cr1^i^	40.23 (3)	N2—C8—H8A	109.1
N3—Cr1—Cr1^i^	141.77 (4)	C9—C8—H8A	109.1
N2—Cr1—Cr1^i^	122.99 (4)	N2—C8—H8B	109.1
C9—O1—Cr1	109.12 (8)	C9—C8—H8B	109.1
C9—O1—Cr1^i^	127.69 (9)	H8A—C8—H8B	107.8
Cr1—O1—Cr1^i^	99.30 (5)	O1—C9—C8	108.09 (12)
C1—O2—Cr1	128.37 (9)	O1—C9—H9A	110.1
C1—O2—Mn1	116.79 (9)	C8—C9—H9A	110.1
Cr1—O2—Mn1	114.84 (4)	O1—C9—H9B	110.1
C4—O3—Mn1	118.54 (9)	C8—C9—H9B	110.1
C4—O3—H3	109.4	H9A—C9—H9B	108.4
Mn1—O3—H3	132.1	N2—C10—H10A	109.5
C6—O4—Cr1	117.74 (9)	N2—C10—H10B	109.5
C6—O4—Mn1^i^	126.56 (9)	H10A—C10—H10B	109.5
Cr1—O4—Mn1^i^	115.19 (5)	N2—C10—H10C	109.5
C5—N1—C2	110.85 (12)	H10A—C10—H10C	109.5
C5—N1—C3	110.47 (13)	H10B—C10—H10C	109.5
C2—N1—C3	110.54 (13)	N3—C11—S1	179.83 (19)
C5—N1—Mn1	112.29 (10)	N4—C12—S2	179.54 (18)
C2—N1—Mn1	104.53 (9)	O5—C13—N5	124.30 (17)
C3—N1—Mn1	107.99 (9)	O5—C13—H13A	117.9
C10—N2—C7	108.96 (12)	N5—C13—H13A	117.9
C10—N2—C8	109.64 (12)	N5—C14—H14A	109.5
C7—N2—C8	111.83 (12)	N5—C14—H14B	109.5
C10—N2—Cr1	115.34 (10)	H14A—C14—H14B	109.5
C7—N2—Cr1	104.72 (9)	N5—C14—H14C	109.5
C8—N2—Cr1	106.32 (9)	H14A—C14—H14C	109.5
C11—N3—Cr1	165.05 (14)	H14B—C14—H14C	109.5
C12—N4—Mn1	171.08 (13)	N5—C15—H15A	109.5
C13—N5—C15	121.22 (15)	N5—C15—H15B	109.5
C13—N5—C14	121.02 (15)	H15A—C15—H15B	109.5
C15—N5—C14	117.73 (14)	N5—C15—H15C	109.5
C16—N6—C17	120.90 (15)	H15A—C15—H15C	109.5
C16—N6—C18	121.30 (15)	H15B—C15—H15C	109.5
C17—N6—C18	117.37 (14)	O6—C16—N6	126.22 (17)
O2—C1—C2	109.56 (12)	O6—C16—H16A	116.9
O2—C1—H1A	109.8	N6—C16—H16A	116.9
C2—C1—H1A	109.8	N6—C17—H17A	109.5
O2—C1—H1B	109.8	N6—C17—H17B	109.5
C2—C1—H1B	109.8	H17A—C17—H17B	109.5
H1A—C1—H1B	108.2	N6—C17—H17C	109.5
N1—C2—C1	110.99 (13)	H17A—C17—H17C	109.5
N1—C2—H2A	109.4	H17B—C17—H17C	109.5
C1—C2—H2A	109.4	N6—C18—H18A	109.5
N1—C2—H2B	109.4	N6—C18—H18B	109.5
C1—C2—H2B	109.4	H18A—C18—H18B	109.5
H2A—C2—H2B	108	N6—C18—H18C	109.5
N1—C3—C4	110.37 (15)	H18A—C18—H18C	109.5
N1—C3—H3A	109.6	H18B—C18—H18C	109.5
C4—C3—H3A	109.6		
			
O2—Cr1—O1—C9	−126.82 (9)	O3—Mn1—N1—C3	−24.90 (10)
O4—Cr1—O1—C9	51.49 (9)	O2—Cr1—N2—C10	−33.38 (11)
O1^i^—Cr1—O1—C9	135.43 (11)	O4—Cr1—N2—C10	148.52 (11)
N2—Cr1—O1—C9	−29.49 (9)	O1—Cr1—N2—C10	−117.11 (10)
Cr1^i^—Cr1—O1—C9	135.43 (11)	O1^i^—Cr1—N2—C10	−161.80 (12)
O2—Cr1—O1—Cr1^i^	97.75 (5)	N3—Cr1—N2—C10	59.00 (11)
O4—Cr1—O1—Cr1^i^	−83.93 (5)	Cr1^i^—Cr1—N2—C10	−128.72 (9)
O1^i^—Cr1—O1—Cr1^i^	0	O2—Cr1—N2—C7	−153.11 (9)
N2—Cr1—O1—Cr1^i^	−164.92 (5)	O4—Cr1—N2—C7	28.78 (9)
O1—Cr1—O2—C1	173.65 (13)	O1—Cr1—N2—C7	123.16 (9)
O1^i^—Cr1—O2—C1	−106.42 (13)	O1^i^—Cr1—N2—C7	78.47 (15)
N3—Cr1—O2—C1	−4.47 (13)	N3—Cr1—N2—C7	−60.73 (9)
N2—Cr1—O2—C1	90.32 (13)	Cr1^i^—Cr1—N2—C7	111.54 (8)
Cr1^i^—Cr1—O2—C1	−146.32 (12)	O2—Cr1—N2—C8	88.37 (10)
O1—Cr1—O2—Mn1	−5.92 (5)	O4—Cr1—N2—C8	−89.74 (9)
O1^i^—Cr1—O2—Mn1	74.01 (6)	O1—Cr1—N2—C8	4.64 (9)
N3—Cr1—O2—Mn1	175.97 (6)	O1^i^—Cr1—N2—C8	−40.06 (18)
N2—Cr1—O2—Mn1	−89.25 (6)	N3—Cr1—N2—C8	−179.25 (10)
Cr1^i^—Cr1—O2—Mn1	34.11 (5)	Cr1^i^—Cr1—N2—C8	−6.98 (11)
O4^i^—Mn1—O2—C1	121.25 (11)	O2—Cr1—N3—C11	87.0 (5)
N4—Mn1—O2—C1	−100.05 (11)	O4—Cr1—N3—C11	−91.3 (5)
O3—Mn1—O2—C1	30.08 (14)	O1^i^—Cr1—N3—C11	−175.8 (5)
N1—Mn1—O2—C1	3.21 (11)	N2—Cr1—N3—C11	−9.9 (5)
O4^i^—Mn1—O2—Cr1	−59.13 (6)	Cr1^i^—Cr1—N3—C11	−179.4 (5)
N4—Mn1—O2—Cr1	79.57 (6)	Cr1—O2—C1—C2	152.10 (10)
O3—Mn1—O2—Cr1	−150.30 (6)	Mn1—O2—C1—C2	−28.33 (16)
N1—Mn1—O2—Cr1	−177.17 (6)	C5—N1—C2—C1	76.39 (16)
O4^i^—Mn1—O3—C4	−121.36 (13)	C3—N1—C2—C1	−160.77 (13)
N4—Mn1—O3—C4	105.78 (13)	Mn1—N1—C2—C1	−44.81 (14)
O2—Mn1—O3—C4	−28.96 (16)	O2—C1—C2—N1	50.17 (17)
N1—Mn1—O3—C4	−1.57 (12)	C5—N1—C3—C4	−74.85 (17)
O1—Cr1—O4—C6	−91.82 (10)	C2—N1—C3—C4	162.08 (13)
O1^i^—Cr1—O4—C6	−172.13 (10)	Mn1—N1—C3—C4	48.29 (15)
N3—Cr1—O4—C6	86.19 (10)	Mn1—O3—C4—C3	27.02 (18)
N2—Cr1—O4—C6	−8.37 (10)	N1—C3—C4—O3	−49.63 (19)
Cr1^i^—Cr1—O4—C6	−132.03 (10)	Cr1—O4—C6—C7	−14.59 (15)
O1—Cr1—O4—Mn1^i^	80.51 (5)	Mn1^i^—O4—C6—C7	174.07 (9)
O1^i^—Cr1—O4—Mn1^i^	0.19 (5)	C10—N2—C7—C6	−168.24 (14)
N3—Cr1—O4—Mn1^i^	−101.49 (6)	C8—N2—C7—C6	70.40 (16)
N2—Cr1—O4—Mn1^i^	163.96 (6)	Cr1—N2—C7—C6	−44.32 (14)
Cr1^i^—Cr1—O4—Mn1^i^	40.29 (5)	O4—C6—C7—N2	39.62 (17)
O4^i^—Mn1—N1—C5	176.21 (10)	C10—N2—C8—C9	145.05 (14)
N4—Mn1—N1—C5	11.66 (11)	C7—N2—C8—C9	−93.99 (15)
O2—Mn1—N1—C5	−97.53 (11)	Cr1—N2—C8—C9	19.74 (15)
O3—Mn1—N1—C5	97.14 (11)	Cr1—O1—C9—C8	47.36 (14)
O4^i^—Mn1—N1—C2	−63.56 (10)	Cr1^i^—O1—C9—C8	166.30 (9)
N4—Mn1—N1—C2	131.89 (10)	N2—C8—C9—O1	−44.64 (17)
O2—Mn1—N1—C2	22.71 (9)	C15—N5—C13—O5	0.6 (3)
O3—Mn1—N1—C2	−142.63 (10)	C14—N5—C13—O5	178.61 (17)
O4^i^—Mn1—N1—C3	54.17 (11)	C17—N6—C16—O6	3.1 (3)
N4—Mn1—N1—C3	−110.38 (11)	C18—N6—C16—O6	175.29 (18)
O2—Mn1—N1—C3	140.44 (11)		

Symmetry codes: (i) −*x*+1, −*y*, −*z*+1.

### Hydrogen-bond geometry (Å, °)

**Table:**

*D*—H···*A*	*D*—H	H···*A*	*D*···*A*	*D*—H···*A*
O3—H3···O5	0.82	1.78	2.5985 (18)	176
